# DUNet: a novel dehazing model based on outdoor images

**DOI:** 10.3389/fpls.2025.1632052

**Published:** 2025-10-07

**Authors:** Wei Zhao, Qiusheng Zhang, Mingliang Li, Guanshi Ye, Zichen Liu, Mingyang Qi, Helong Yu, You Tang

**Affiliations:** ^1^ School of Electrical and Information Engineering, Jilin Agricultural Science and Technology University, Jilin, China; ^2^ College of Underwater Acoustic Engineering, Harbin Engineering University, Harbin, China; ^3^ College of Information Technology, Jilin Agricultural University, Changchun, China

**Keywords:** image dehazing, deep learning, image processing, smart agriculture, outdoor images

## Abstract

Image dehazing technology is widely utilized in outdoor environments, especially in precision agriculture, where it enhances image quality and monitoring accuracy. However, conventional dehazing methods have exhibited limited performance in complex outdoor conditions, necessitating the development of more advanced models to address these challenges. This paper proposes DUNet, a high-performance image dehazing model that is well-suited for outdoor smart agriculture applications. In this study, we first introduce a novel hybrid convolution block, MixConv, designed to fully extract detailed feature information from images. Secondly, by incorporating the atmospheric scattering model, we propose a dehazing feature extraction unit, DFEU, integrated between the encoder and decoder, to establish a mapping relationship between hazy and haze-free images in the feature space. Finally, the SK fusion mechanism dynamically fuses feature maps extracted from multiple paths. To evaluate the dehazing performance of DUNet, we constructed a dataset consisting of 1,978 pairs of hazy UAV images of paddy fields. DUNet achieved a PSNR of 36.0206 and an SSIM of 0.9946 on this dataset. We further validated DUNet’s performance on a remote sensing dataset, achieving a PSNR of 37.2887 and an SSIM of 0.9933. Experimental results demonstrate that, compared to other well-established image dehazing models, DUNet offers superior performance, confirming its potential and feasibility for outdoor smart agriculture dehazing tasks.

## 1 Introduction

With the continuous advancement of social and technological progress, smart agriculture has emerged as a critical direction for modern agricultural development, rapidly gaining popularity and widespread application. In modern smart agriculture, drone technology serves as an essential tool for precision farming, widely employed in areas such as farmland monitoring, crop growth evaluation, pest detection, and soil moisture analysis (Guo et al., 2025; Su et al., 2024; Zhao et al., 2025). UAVs, equipped with high-resolution cameras, infrared sensors, and multispectral imaging devices, can efficiently cover extensive agricultural areas and capture real-time images of farmland, thereby providing precise decision support for agricultural management (Yang et al., 2025). By integrating deep learning and computer vision technologies, drone systems can not only assess crop growth conditions with high accuracy but also detect issues such as pests, water scarcity, or nutrient deficiencies in a timely manner, thereby significantly enhancing the intelligence and automation of farmland management (Wen et al., 2024). The widespread adoption of this technology has transformed agricultural production from traditional, experience-based management to data-driven, precision-based models, enhancing crop yields, reducing production costs, and minimizing pesticide use, thereby promoting sustainable agricultural development. However, drones still face several challenges in practical applications, particularly under complex weather conditions such as haze, which degrades image quality. Specifically, haze conditions result in issues such as insufficient contrast and blurred details in images captured by drones, hindering the accurate assessment of crop health and timely identification of pests and diseases. This imposes substantial limitations on the effectiveness of drone applications, particularly in large-scale farmland monitoring, where low-quality images can lead to decreased recognition accuracy and even undermine the intelligence of agricultural production. Therefore, it is essential to restore or enhance the captured blurred images to ensure the usability and reliability of the data, facilitating the smooth execution of subsequent detection and identification tasks. This will not only improve the accuracy and system stability of drone-based farmland monitoring but also play a crucial role in advancing the intelligent development of agriculture (Joshi et al., 2024; Yu et al., 2024b).

Image dehazing technology is a crucial task in computer vision, aimed at restoring hazy images to clear and visible ones. The presence of haze or other factors often leads to the loss of image details and reduced contrast, which in turn affects subsequent image analysis and processing. Dehazing technology can effectively mitigate or eliminate these degradations, restoring image clarity and detail and making the image more vivid and informative. This not only enhances the visual experience but also provides more accurate data for subsequent advanced visual tasks, such as object detection and target recognition, thereby aiding in the extraction of more valuable information from images (Goyal et al., 2024).In the early stages of image dehazing research, the physical processes behind haze formation were not yet understood. Most methods relied on image enhancement techniques to achieve deblurring, such as histogram equalization (Dale-Jones and Tjahjadi, 1993; Kim et al., 2001; Zhu et al., 2013), color correctio (Huang et al., 2014), and others. Following the introduction of the atmospheric scattering model (Nayar and Narasimhan, 1999), researchers recognized that hazy images result from the degradation of clear images and began developing dehazing algorithms based on this model. The atmospheric scattering model is represented by Equation 1:


(1) I(x)=J(x)t(x)+A(1−t(x))

Here, I is the hazy image, J is the clear image, A is the global atmospheric light, t is the transmission map, and x is the pixel index. When the global atmospheric light A is uniform, t is expressed as Equation 2:


(2) $$t\left(x\right)=e^{-\beta d\left(x\right)}$$

Where β is the scattering coefficient of the atmosphere and d is the depth information.

Early image dehazing methods based on the atmospheric scattering model, predominantly relied on prior knowledge. In 2009, He et al. proposed the classic dark channel prior (DCP) algorithm (He et al., 2009), which posits that most local regions in haze-free outdoor images contain pixels with very low intensity in at least one color channel. By integrating the atmospheric scattering model, DCP can generate effective depth maps and restore clear images. However, the DCP method has limitations, primarily due to its reliance on statistical priors, which may not be suitable for images where the atmospheric light closely resembles the scene objects. In 2015, Zhu et al. proposed the color attenuation prior (CAP) (Zhu et al., 2015), which models the scene depth in hazy images using a linear model, recovers depth information through supervised learning methods, and then estimates the transmission rate using the atmospheric scattering model to obtain the dehazed image. However, due to the limited learning capacity of the model, many prior methods still exhibit shortcomings in representation and accuracy.

With the rapid development of deep learning technologies, many end-to-end convolutional neural networks (CNNs) have been employed by researchers for image dehazing tasks (Jackson et al., 2024). CNNs reduce the reliance on manually designed priors by automatically learning useful image features. For example, Cai et al. proposed DehazeNet (Cai et al., 2016), which takes hazy images as input, outputs the medium transmission map, and then uses the atmospheric scattering model to recover the clear image. Li et al. proposed AOD-Net (Li et al., 2017), which is based on a redesigned atmospheric scattering model and directly generates clear images through CNNs, eliminating the need to estimate the transmission matrix and atmospheric light. Song et al. proposed a compact dehazing model, gUNet (Song et al., 2022), which introduces minimal modifications to U-Net (Ronneberger et al., 2015) and incorporates residual blocks with a gating mechanism. This not only reduces the model’s parameter count effectively but also yields good dehazing results. The DCPDN dehazing model (Zhang and Patel, 2018) proposed by Zhang et al. integrates the atmospheric scattering model into the network to optimize the learning of the transmission map, atmospheric light, and dehazed image, and introduces Generative Adversarial Networks (Goodfellow et al., 2014) to enhance details, significantly improving dehazing performance. Chen et al. proposed the end-to-end gated context aggregation dehazing network GCANet (Chen et al., 2019), which introduces a smooth dilation technique to eliminate grid artifacts and utilizes a gating network to fuse features across different levels, achieving better dehazing results. Although these methods have advanced image dehazing technology, they still face challenges in handling extreme weather conditions, low-quality images, and real-time scenes. Nevertheless, the introduction of end-to-end networks has undeniably accelerated the progress of image dehazing research.

In recent years, researchers have further enhanced the dehazing performance of models by incorporating attention mechanisms. For example, Xu et al. proposed a novel feature attention (FA) mechanism that combines channel and pixel attention, which was applied to CNNs to expand the network’s representational capacity. Through local residual learning, the FFA-Net (Qin et al., 2020) network can better focus on learning effective information. Chen et al. proposed the DEA-Net (Chen et al., 2023), which is based on detail-enhancing convolution and content-guided attention. This model introduces differential convolution to enhance representational capacity and integrates three attention mechanisms to comprehensively extract image detail features. With the widespread application of Transformers (Vaswani et al., 2017) in computer vision, and inspired by the Vision Transformer (Dosovitskiy et al., 2020), Song et al. proposed DehazeFormer (Song et al., 2023). This model employs a shifted window partitioning scheme with reflective padding and integrates a convolutional spatial information aggregation scheme parallel to attention. Experimental results demonstrate its excellent performance in dehazing tasks. Guo et al. combined Transformers with CNNs to propose the Dehamer (Guo et al., 2022) model, which retains the advantages of Transformer in global context modeling while preserving CNN’s capability in local representations, thereby significantly improving dehazing performance. However, some color bias persists in its color restoration, causing the dehazed images to differ in color from the clear images.

Although end-to-end networks have improved performance within physical model constraints, they are often confined to the original image space and fail to fully utilize the physical information in the feature space. Therefore, Dong et al. proposed a physics-based dehazing network, PFDN (Dong and Pan, 2020), which explicitly utilizes the physical model in the feature space by introducing a key component, the ASM-based Feature Dehazing Unit (FDU), learning the required useful information to achieve more effective dehazing. However, the FDU overlooks the fact that atmospheric light and transmission maps are not always uniform, and their features cannot be approximated similarly. To accurately implement the physical model in the deep network feature space, Zheng et al. proposed a physically-aware dual-branch unit (PDU) (Zheng et al., 2023), which separately captures features corresponding to atmospheric light and transmission maps in two branches, considering the physical properties of each factor. This allows for more precise synthesis of potential clear image features based on the physical model and facilitates information transfer and feature extraction in the feature space.

In recent years, dehazing research based on deep learning has garnered increasing attention. Li et al. proposed an efficient dehazing method applicable to both outdoor and remote sensing images, which integrates the strengths of image enhancement and image restoration techniques (Li et al., 2023). Experimental results on both synthetic and real-world datasets demonstrated that this method outperformed existing approaches. After that, Li et al. further introduced UAVD-Net, a novel dehazing framework tailored for drone-based remote sensing images affected by spatially varying haze (Li et al., 2025). UAVD-Net employs both global and local feature extraction mechanisms to effectively eliminate non-uniform haze across spatial regions, consistently achieving superior performance compared to state-of-the-art methods on diverse datasets. Similarly, Cui et al. proposed an image dehazing network called EENet, which aims to achieve image dehazing through enhanced spatial-spectral learning (Cui et al., 2025). This method works through the coordinated efforts of three modules: frequency processing, spatial processing, and dual-domain interaction. Based on modelling global dependencies and multi-scale features, it achieves information fusion between the frequency domain and the spatial domain to improve image dehazing effects, and has achieved state-of-the-art performance on synthetic and real-world image dehazing datasets. However, due to the domain gap between synthetic and real images, models trained solely on synthetic data often lack generalization in real-world scenarios. To overcome this limitation, Su et al. proposed DNMGDT, a dehazing network that integrates multi-prior guidance with domain transfer mechanisms (Su et al., 2025). By leveraging pseudo-label supervision, adaptive weighting, and physically guided domain transfer strategies, DNMGDT significantly improves performance on real-world hazy images. Collectively, these deep learning–based dehazing approaches offer valuable insights and advancements for the field of image restoration.

Smart agriculture plays a vital role in modern society, and farmland monitoring, as a key component, significantly contributes to its advancement through efficient and intelligent management. In farmland monitoring, the images collected are often influenced by the complexity of outdoor weather conditions, such as fog, causing drones to capture blurry images with visible haze. This impacts subsequent evaluation and recognition tasks, making it challenging to accurately identify and analyze targets. Hazy images not only reduce the accuracy of drone-based farmland monitoring systems but may also adversely impact agricultural decision-making, thereby affecting the responsiveness and efficiency of farmland management (Qiu et al., 2024; Zhang et al., 2022). To address this issue, image dehazing technology plays a crucial role in smart agriculture by effectively enhancing the clarity and detail of images, thus providing reliable visual support for tasks such as crop monitoring and pest detection. However, existing image dehazing algorithms continue to suffer from poor performance, primarily due to their inability to directly establish the relationship between hazy and clear images in the feature space, leading to insufficient utilization of physical image information and poor restoration quality. To address this, we propose a novel image dehazing model, DUNet, based on the atmospheric scattering model, designed to fully extract dehazing features and effectively restore visual information affected by haze and other environmental factors. Specifically, we utilize the classic U-Net architecture with residual connections as the backbone to extract multi-scale information. Next, we employ the hybrid convolution module, MixConv, which incorporates depthwise separable convolution and multi-scale gated convolution, to thoroughly extract detailed feature information. Furthermore, we integrate a dehazing feature extraction unit based on the atmospheric scattering model into the network, which predicts atmospheric light and transmission maps through dual paths, establishing the relationship between hazy and clear images in the feature space. Finally, we utilize the SK fusion module (Song et al., 2022) to dynamically merge the feature maps extracted from different paths. Our main contributions are as follows:

1. Based on real UAV-collected rice field image data, a haze-affected paddy field image dataset was synthesized using the atmospheric scattering model.
2. A new end-to-end dehazing model, DUNet, for smart agriculture is proposed based on the atmospheric scattering model.
3. A hybrid convolution module, MixConv, containing depthwise separable convolution and multi-scale gated convolution, is proposed to enhance the model’s ability to extract multi-scale information.
4. A dehazing feature extraction unit (DFEU) is proposed to establish the relationship between hazy and clear images in the feature space.
5. Experimental results show that DUNet performs well in dehazing tasks on the remote sensing haze dataset and rice field haze image dataset, demonstrating good robustness and providing a new strategy for image dehazing.

## 2 Materials and methods

### 2.1 Datasets

**Figure 1:**
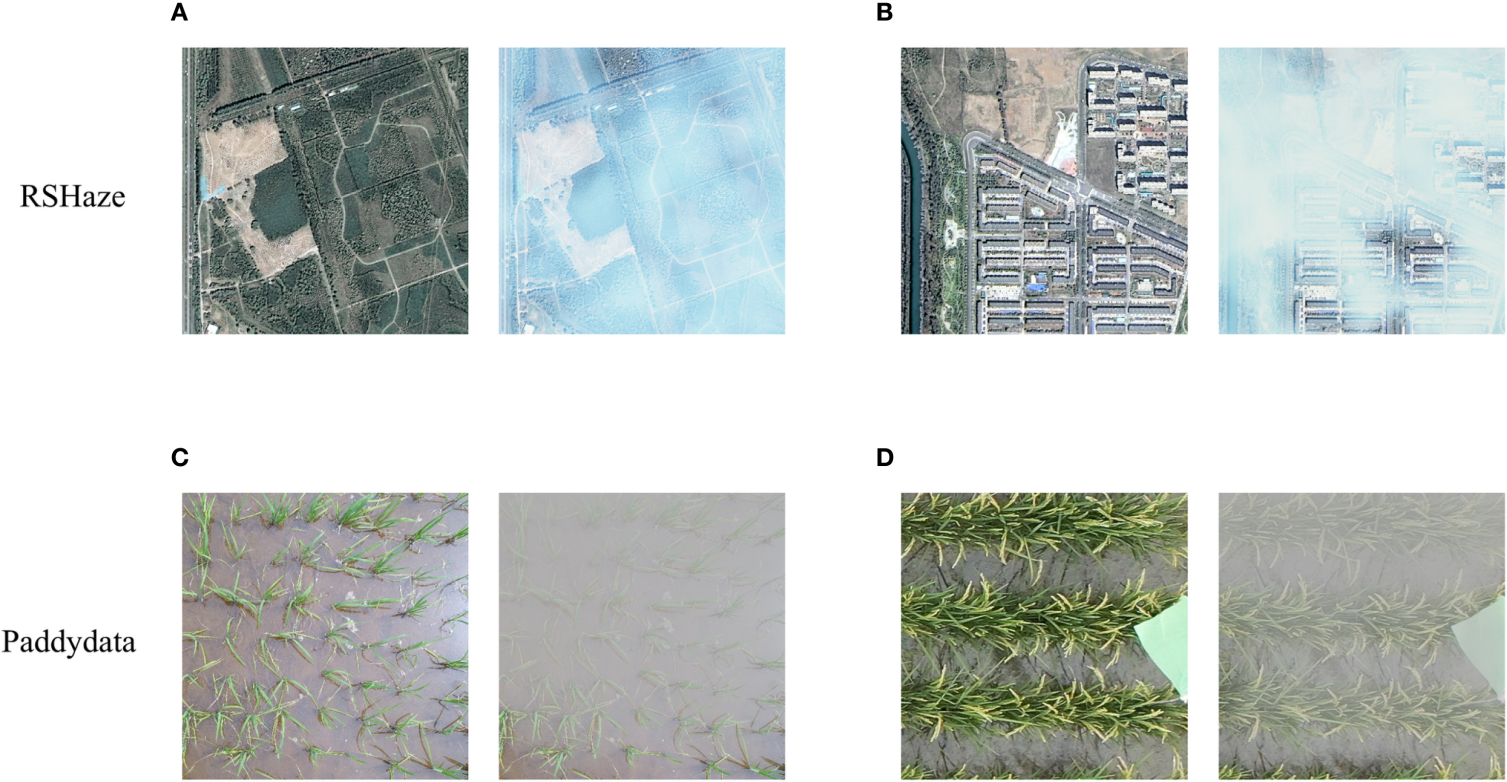
Images of RSHaze and Paddydata datasets. **(A)** and **(B)** depict images of varying haze concentrations from RSHaze, while **(C)** and **(D)** show haze images of different rice paddies from Paddydata.

This study employed two datasets, one of which is the publicly available remote sensing dataset, RSHaze (Lihe et al., 2024). Due to the highly uneven distribution of haze in remote sensing images, haze removal is commonly considered a classic non-uniform image dehazing problem. Therefore, the dehazing performance of the proposed model was evaluated on the remote sensing dataset. The RSHaze dataset comprises 1330 pairs of remote sensing images, each resized to 512x512 pixels. As per the official split, 1000 pairs are designated for training, and the remaining 330 pairs are allocated for testing. The second dataset is derived from two paddy field datasets, URC (Bai et al., 2023) and DPRD (Ye et al., 2024). The images are cropped to 512x512 pixels, resulting in a total of 1978 clear paddy field images. According to Equations 1, 2, after accurately estimating the depth information of the image, the blurred image can be synthesized using the atmospheric scattering model. Thus, we first estimate the depth information of the clear paddy field images using Monodepth2 (Godard et al., 2018). Next, following Equation 2, the scattering coefficient is set to 2.0 to compute the transmission map. Finally, based on Equation 1, the atmospheric light is set to 170 to obtain the synthesized blurred image. Ultimately, we constructed a haze image dataset for paddy fields, named Paddydata. Paddydata consists of 1978 pairs of paddy field images, each resized to 512x512 pixels. The dataset is randomly split into training, validation, and test sets with a 7:1:2 ratio: 1385 pairs for training, 197 pairs for validation, and 396 pairs for testing. The paired images from the two datasets are shown in **Figures 1A, B** are randomly selected images with varying haze concentrations from RSHaze, while **Figures 1C, D** are paired images from different paddy fields. During training, to increase data diversity, we applied five data augmentation techniques: random cropping, random horizontal flipping, random rotation, aligned cropping, and pixel normalization. Specifically, the images are randomly cropped to 256x256 pixels, with a 50% chance of horizontal flipping. The rotation angles are randomly chosen from 0°, 90°, 180°, or 270°. Center cropping is applied to align the image size, and pixel values are normalized to the range [-1,1]. These data augmentation techniques effectively enhanced data diversity, mitigated overfitting to specific sample features, and improved the robustness and generalization ability of the model. The dataset is publicly available at (https://github.com/MaiheZHao/data).

### 2.2 Network architecture

**Figure 2:**
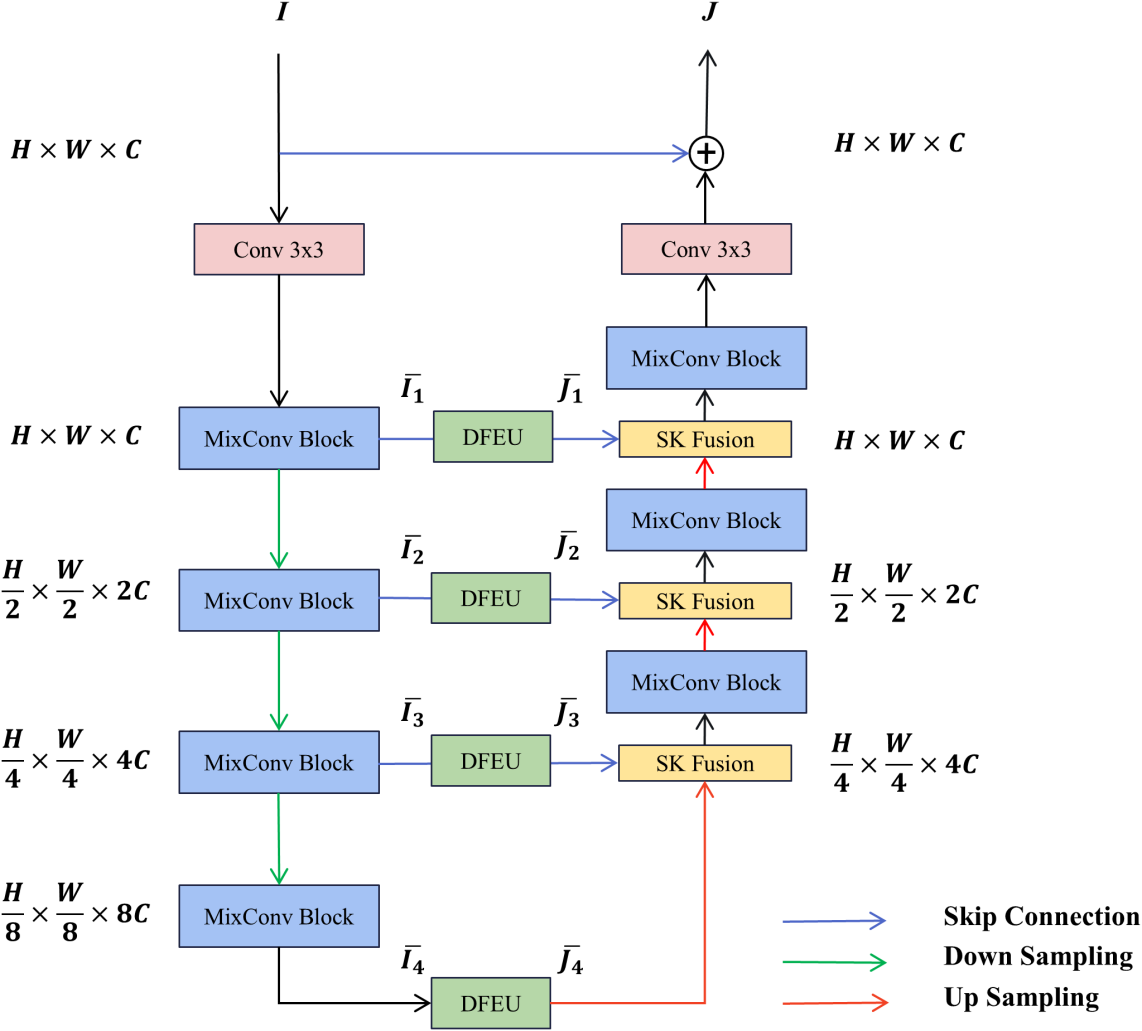
Overall structure of DUNet.

The DUNet model utilizes the MixConv module for feature extraction at every stage. A dehazing feature extraction unit (DFEU), based on the atmospheric scattering model, is inserted between the encoder and decoder to extract fog-free features. The decoder employs the SK fusion module to dynamically combine feature representations from multiple paths. The overall architecture of DUNet is depicted in **Figure 2**. The blurry image I first passes through the MixConv module and a downsampling encoder to extract multi-scale blurry image features $\bar{I_{i}}$ within the feature space, where i is 1, 2, 3, or 4. Subsequently, the dehazing feature extraction unit extracts fog-free features $\bar{J_{i}}$ , where i is 1, 2, 3, or 4. Subsequently, the SK fusion module combines the feature maps extracted by the encoder’s downsampling and those upsampled and restored by the decoder. The MixConv module decodes the fog-free features into a final dehazed image. Finally, a global residual operation is applied to the blurry image I to produce the final dehazed image J .

### 2.3 MixConv block

**Figure 3:**
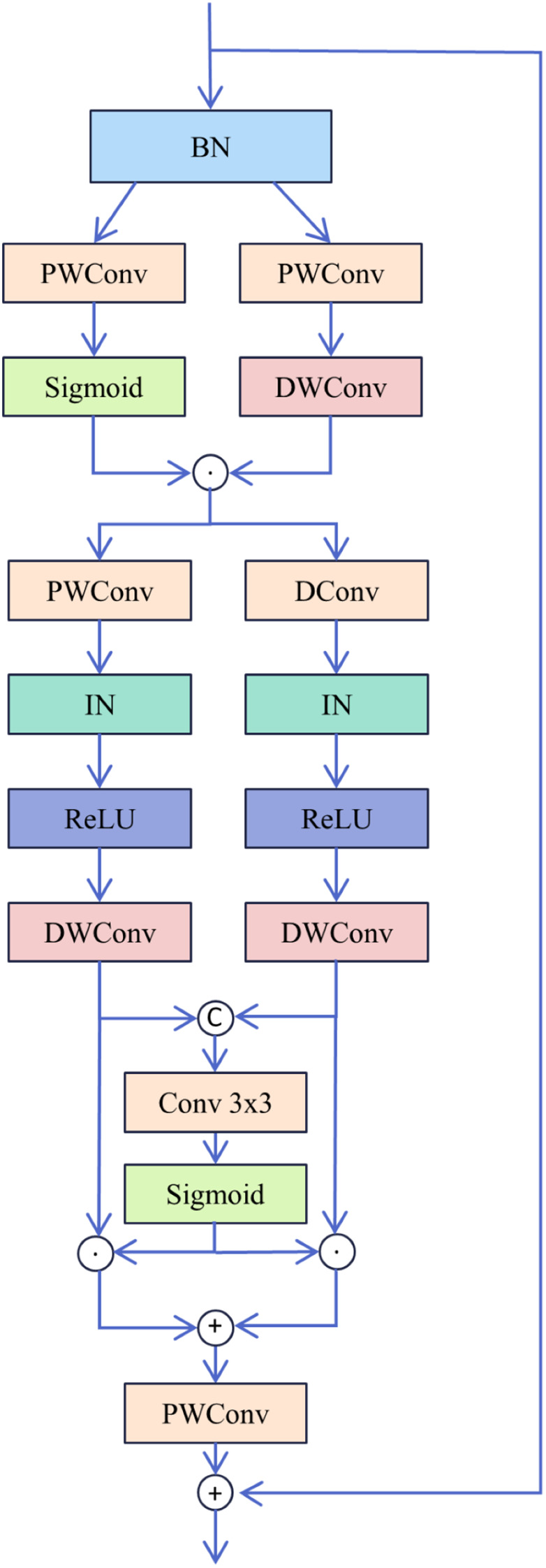
Structure of MixConv Block.


The MixConv Block primarily utilizes depthwise separable convolutions (Chollet, 2017) and a multi-scale gated fusion mechanism,and the structure of the MixConv Block is depicted in **Figure 3**. First, the input feature x is normalised via BatchNorm (Ioffe and Szegedy, 2015) to enhance network efficiency and stability, yielding feature $\bar{x}$ . Subsequently, $\bar{x}$ undergoes dual-path convolution processing.One branch employs deep separable convolution to efficiently extract local features, while the other branch incorporates a gating mechanism using the Sigmoid function to generate channel weights for the features, thereby enhancing the network’s expressive capacity. This results in the intermediate feature $x_{1}$ . Second, the intermediate feature $x_{1}$ undergoes dual-path convolution processing. One branch employs deep separable convolution to further extract local features $x_{2}$ , while the other branch utilises dilated convolution (Yu and Koltun, 2015) and deep convolution to extract features $x_{3}$ within a larger receptive field. Both branches incorporate InstanceNorm (Ulyanov et al., 2016) and the ReLU activation function to achieve normalisation and non-linear enhancement. Finally, a gating mechanism fuses the features from the dual-path convolutions. The outputs $x_{2}$ and $x_{3}$ are concatenated, then convolved with the Sigmoid function to generate channel weights $w_{1}$ and $\;w_{2}$ for each feature. These weighted features are subsequently fused, followed by a pointwise convolution for channel mapping to unify dimensions. The result is connected via residual connections to the original input feature x , yielding the output feature y . With its computational process described by Equations 3–8. Here, PWConv denotes pointwise convolution, DWConv denotes depthwise convolution, and DConv denotes dilated convolution.

(3) $$\bar{x}=BatchNorm\left(x\right)$$

(4) $$x_{1}=Sigmoid\left(PWConv\left(\bar{x}\right)\right)*DWConv\left(PWConv\left(\bar{x}\right)\right)$$

(5) $$x_{2}=DWConv\left(ReLU\left(InstanceNorm\left(PWConv\left(x_{1}\right)\right)\right)\right)$$

(6) $$x_{3}=DWConv\left(ReLU\left(InstanceNorm\left(DConv\left(x_{1}\right)\right)\right)\right)$$

(7) $$\left(w_{1},w_{2}\right)=Sigmoid\left(Conv\left(Concatenate\left(x_{2},x_{3}\right)\right)\right)$$

(8) $$y=x+PWconv\left(w_{1}*x_{2}+w_{2}*x_{3}\right)$$

### 2.4 Dehazing feature extraction unit

The atmospheric scattering model is commonly employed to describe the transition of a clear image to a hazy image. Due to the uncertainties in atmospheric light and the transmission map, haze removal from real hazy images remains a central focus for researchers. Methods that directly estimate atmospheric light and the transmission map in the original space may result in error accumulation. Inspired by FDU and PDU, incorporating physical priors into the feature space ensures the model aligns with the atmospheric scattering model, thereby enhancing the interpretability of the dehazing process while mitigating the impact of estimation errors in atmospheric light and the transmission map. This study introduces a novel dehazing feature extraction unit, DFEU, which predicts atmospheric light and the transmission map through a dual-path mechanism, establishing a relationship between hazy and dehazed images in the feature space, and synthesizing the features of the potential clear image with greater accuracy based on the physical model. First, we redefine the atmospheric scattering model, as shown in Equations 9, 10.


(9) $$J\left(x\right)=I\left(x\right)\frac{1}{t\left(x\right)}+A\left(1-\frac{1}{t\left(x\right)}\right)$$

(10) $$J\left(x\right)=\left(I\left(x\right)-A\right)\frac{1}{t\left(x\right)}+A$$

Then, features are extracted through the kernel K, and Equation 9 can be expressed as Equation 11:


(11) $$k⊛J=k⊛\left(\left(I-A\right)⊙\frac{1}{t}\right)+k⊛A$$

where ⊛ represents the convolution operator, and ⊙ represents the Hadamard product. We then introduce K , J , I , A and D as matrix-vector forms of k , J , I , A , and $\frac{1}{t}$ , as shown in Equation 12. We can compute the formula through algebraic operations. Additionally, the diagonal vectors of the diagonal matrix D correspond to the vectorization of $\frac{1}{t}$ .


(12) KJ=K(I−A)D+KA=KDI−KDA+KA

Next, we decompose the matrix KD into the product of two matrices K and Q , as indicated in Equation 13.


(13) KJ=Q(KI)−Q(KA)+KA

We can denote $\bar{A}$ as an approximation of the atmospheric light corresponding to the feature KA and $\bar{t}$ as an approximation of the transmission map corresponding to the feature $Q^{-1}$ . KI and KJ can be considered the extracted features $\bar{I}$ and $\bar{J}$ of the hazy image  I and its corresponding clear image J , respectively. Therefore, based on Equation 12, we can calculate the physically perceived features, as shown in Equation 14.


(14) $$\bar{J}=\left(\bar{I}-\bar{A}\right)⊙\frac{1}{\bar{t}}+\bar{A}$$

**Figure 4:**
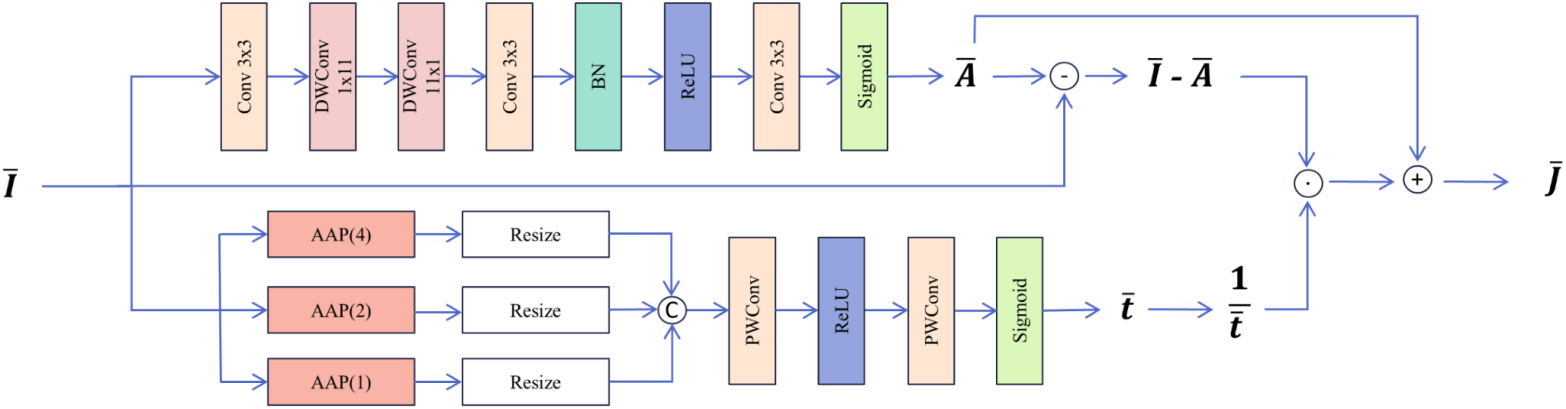
Structure of Dehaze Feature Extraction Unit.


The structure of the DFEU is illustrated in **Figure 4**. The DFEU employs a dual-path design to predict atmospheric light and the transmission map, with one branch generating the atmospheric light $\bar{A}$ . First, local features are extracted via convolution. Subsequently, two shallow striped convolutions with reduced parameters approximate the effect of standard large-kernel depth convolutions, capturing broader contextual information. Next, global context is fused and nonlinear expression enhanced through convolution, BN, and the ReLU activation function. Finally, atmospheric light is extracted via convolutional layer with Sigmoid activation function (Cai et al., 2024). In the other branch, the transmission map $\bar{t}$ is generated. According to prior research, the transmission map is non-uniform. First, we employ a spatial pyramid structure for multi-scale feature extraction, wherein the structure adaptively and uniformly pools the input features across three scales to capture additional feature representations and structural information. Subsequently, we adjust the dimensions of the three outputs and concatenate them to form a one-dimensional attention map (Guo et al., 2020). Next, we employ two points to perform dimensionality reduction and enhance non-linear expression through the convolutional layer and the ReLU activation function. Finally, we utilise the Sigmoid activation function to extract transmission graph features (Yu et al., 2024a), as shown in Equations 15–17. Here, $DWConv^{1\times 11}$ denotes a convolution kernel of (1,11), which emphasizes feature extraction in the horizontal direction, while $DWConv^{11\times 1}$ denotes a convolution kernel of (11,1), which emphasizes feature extraction in the vertical direction.

(15) $$\begin{array}{c} \bar{A}=Sigmoid(Conv(ReLU(BN(Conv(DWConv^{11\times 1}\\ (DWConv^{1\times 11}\left(Conv\left(\bar{I}\right)\right))))))) \end{array}$$

(16) $${\bar{t}}_{1}=Concat\left(Resize\left(\left(AAP\left(1\right)\left(\bar{I}\right),AAP\left(2\right)\left(\bar{I}\right),AAP\left(4\right)\left(\bar{I}\right)\right)\right)\right)$$

(17) $$\bar{t}=Sigmoid\left(PWConv\left(ReLU\left(PWConv\left({\bar{t}}_{1}\right)\right)\right)\right)$$

The proposed DFEU generates dehazed features $\bar{J}$ from the input features $\bar{I}$ , which are subsequently utilized by the decoder to produce dehazed images. DFEU predicts atmospheric light and the transmission map using a dual-path approach, establishing the relationship between hazy and dehazed images in the feature space and synthesizing more accurate features for potential dehazed images.

### 2.5 SK fusion

**Figure 5:**
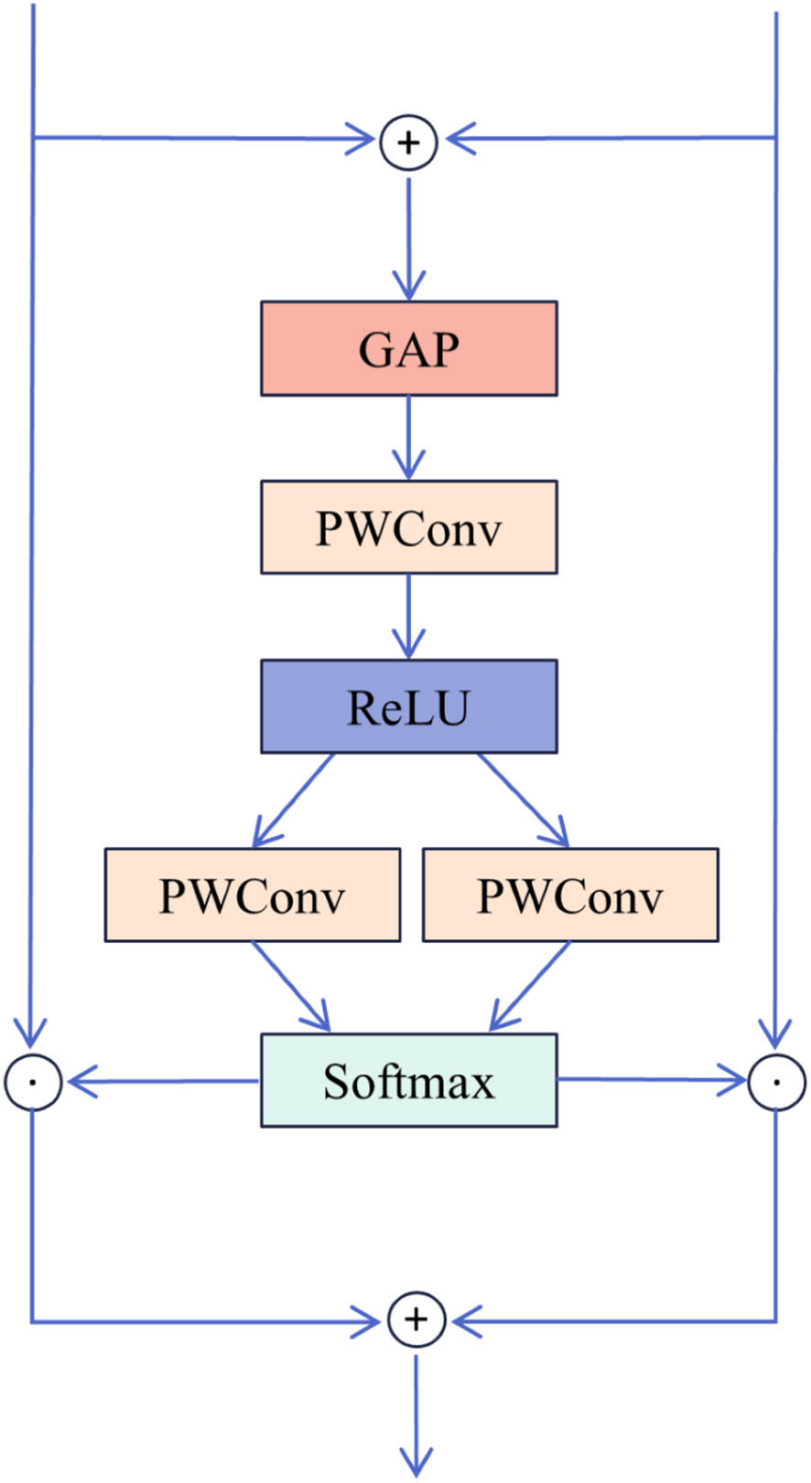
Structure of SK Fusion.


To fuse the dehazed features extracted by DFEU with those from the MixConv module, this study introduces SK Fusion, which is based on the SK module (Li et al., 2019). The structural diagram is presented in **Figure 5**. The two input features consist of the feature map $x_{1}$ from the skip connection and the feature map $x_{2}$ from the main path. Initially, the input features $x_{1}$ and $x_{2}$ are added, followed by global average pooling to extract global information for each channel. Next, the MLP module F comprising two PWConv layers and a ReLU activation function, is introduced to generate a more compact feature representation, thus improving the accuracy of adaptive selection. The two PWConv layers perform dimensionality reduction and expansion, enhancing the efficiency of the MLP module. Finally, the obtained fusion weights are processed using the softmax function and a segmentation operation, enabling the weighted selection of different information, as shown in Equations 18, 19. Ultimately, the fused output feature y is obtained.

(18) $$\left\{w_{1},w_{2}\right\}=Split\left(Softmax\left(F_{mlp}\left(GAP\left(x_{1}+x_{2}\right)\right)\right)\right)$$

(19) $$y=w_{1}*x_{1}+w_{2}*x_{2}$$

### 2.6 Loss function

In this study, the L1 loss function is employed, which quantifies the absolute difference between the predicted and true values, also referred to as Least Absolute Deviation or Absolute Error Loss. In general, it minimizes the sum of the absolute differences between the target values $y_{i}$ and the model’s predicted values $f\left(x_{i}\right)$ . Specifically, let the target value be $y_{i}$ and the model’s predicted value be $f\left(x_{i}\right)$ . The loss function calculation is given by Equation 20.


(20) $$L1loss\left(x,y\right)=\;\frac{1}{n}\sum_{i=1}^{n}\left|y_{i}-f\left(x_{i}\right)\right|$$

## 3 Results and analysis

### 3.1 Experimental environment

In this study, to ensure the objectivity and reliability of the experimental results, all experiments were conducted under a consistent setup. The experiments were conducted on Ubuntu 20.04, utilizing an Intel(R) Xeon(R) Platinum 8352V CPU @ 2.10GHz, paired with an NVIDIA GTX 4090 GPU and 24GB of VRAM. The programming language used was Python 3.8.10, with PyTorch 1.11.0 as the deep learning framework and CUDA 11.3 for GPU acceleration. The training batch size was set to 24, with 1000 epochs. The optimizer used was AdamW (Kingma and Ba, 2014), with an initial learning rate of 0.0002 and a decay factor of 0.01.

### 3.2 Evaluation metrics

This paper employs commonly used image dehazing evaluation metrics, namely Peak Signal-to-Noise Ratio (PSNR) and Structural Similarity (SSIM), to comprehensively evaluate the model’s dehazing performance. PSNR is a metric for image quality that measures the ratio between the maximum signal and background noise. For a grayscale image I of size m×n and a noisy image K, the PSNR calculation formula is provided in Equations 21, 22.


(21) $$MSE=\frac{1}{mn}\sum_{i=0}^{m-1}\sum_{j=0}^{n-1}{\left[I\left(i,j\right)-K\left(i,j\right)\right]}^{2}$$

(22) $$PSNR=10\times lg\left(\frac{MaxValue^{2}}{MSE}\right)$$

Here, MSE represents the Mean Squared Error between two images, and MaxValue refers to the maximum possible pixel value in the image. The minimum value of PSNR is 0, with higher values indicating smaller differences between the two images and less image distortion.

SSIM is a metric used to quantify the structural similarity between two images, based on the human visual system’s sensitivity to changes in local image structures. SSIM evaluates image properties such as brightness, contrast, and structure. Brightness is estimated using the mean, contrast through variance, and structural similarity via covariance. Given two images, x and y , the SSIM calculation formula is provided in Equation 23.


(23) $$SSIM\left(x,y\right)=\frac{\left(2\mu_{x}\mu_{y}+c_{1}\right)\left(2\sigma_{xy}+c_{2}\right)}{\left(\mu_{x}^{2}+\mu_{y}^{2}+c_{1}\right)\left(\sigma_{x}^{2}+\sigma_{y}^{2}+c_{2}\right)}$$

Here, $\mu_{x}$ represents the mean of x , $\sigma_{x}^{2}$ denotes the variance of x , $\mu_{y}$ is the mean of y , $\sigma_{y}^{2}$ is the variance of y  , $\sigma_{xy}$ is the covariance between x and y , $c_{1}={\left(k_{1}L\right)}^{2}$ and $c_{2}={\left(k_{2}L\right)}^{2}$ are constants to maintain stability and avoid division by zero, and L refers to the pixel value range. Typically, $k_{1}$ = 0.01 and $k_{2}$ = 0.03. The minimum value of SSIM is 0, with higher SSIM values indicating greater similarity between the two images.

### 3.3 Ablation experiment

In this study, due to the significant non-uniform distribution of haze in remote sensing images, haze removal is frequently regarded as a classic non-uniform image dehazing problem. Therefore, this study assessed the performance of the proposed model in haze removal using the RSHaze remote sensing dataset. Additionally, the model’s dehazing performance in outdoor agricultural settings was validated using the Paddydata paddy field haze dataset. Through a series of ablation experiments, the performance of each module in the network was tested on both datasets, gradually adding modules to observe their specific effect on network performance. Additionally, we compared the dehazing performance of the proposed DFEU with that of the FDU and PDU models.

#### 3.3.1 Ablation experiment of modules

Based on gUNet, we named the resulting model Model1. We then introduced the MixConv module alone, naming it Model2, followed by the introduction of DFEU alone, resulting in Model3. Finally, both MixConv and DFEU were combined, naming it Model4. The experimental results are presented in **Table 1**. The “√” in the table indicates the inclusion of the module. From **Table 1**, it can be observed that Model1 achieved a PSNR of 33.2461 and an SSIM of 0.9894 on the RSHaze remote sensing dataset, and a PSNR of 34.7628 and an SSIM of 0.9935 on the Paddydata haze image dataset. To enhance the model’s ability to extract multi-scale information, Model2 incorporated the MixConv module, replacing the convolution module of Model1, which leads to a significant improvement in dehazing performance. The number of parameters increased by 2.2371M. For RSHaze, the PSNR increased by 3.4682, and SSIM increased by 0.0035. For Paddydata, PSNR increased by 0.3235, and SSIM increased by 0.0004. Model3 integrated DFEU between the encoder and decoder, directly establishing the relationship between hazy and clear images in the feature space based on the atmospheric scattering model. This further extracts dehazing features from the image and improves the model’s dehazing performance. Compared to Model1, the number of parameters increased by 1.2328M. For RSHaze, the PSNR increased by 1.3134, and the SSIM increased by 0.0015. For Paddydata, PSNR increased by 0.5689, and SSIM increased by 0.0001. Finally, Model4 incorporated both MixConv and DFEU, combining the advantages of both to further improve the model’s performance. Compared to Model1, the number of parameters increased by 3.3493M, and FLOPs increased by 8.4398G. The PSNR and SSIM on RSHaze increased by 4.0426 and 0.0039, reaching values of 37.2887 and 0.9933, respectively. On Paddydata, PSNR and SSIM increased by 1.2578 and 0.0011, reaching values of 36.0206 and 0.9946.

**Table 1:** Ablation experiment results for different modules.

Model	MixConv	DFEU	RSHaze		Paddydata		Parameters/M	FLOPs/G
			PSNR(dB)	SSIM	PSNR(dB)	SSIM		
Model1			33.2461	0.9894	34.7628	0.9935	0.8432	2.8347
Model2	✓		36.7143	0.9929	35.0863	0.9939	3.0803	10.2486
Model3		✓	34.5595	0.9909	35.3317	0.9936	2.0760	3.8639
Model4(ours)	✓	✓	**37.2887**	**0.9933**	**36.0206**	**0.9946**	4.1925	11.2745

The best results are marked bold.

**Figure 6:**
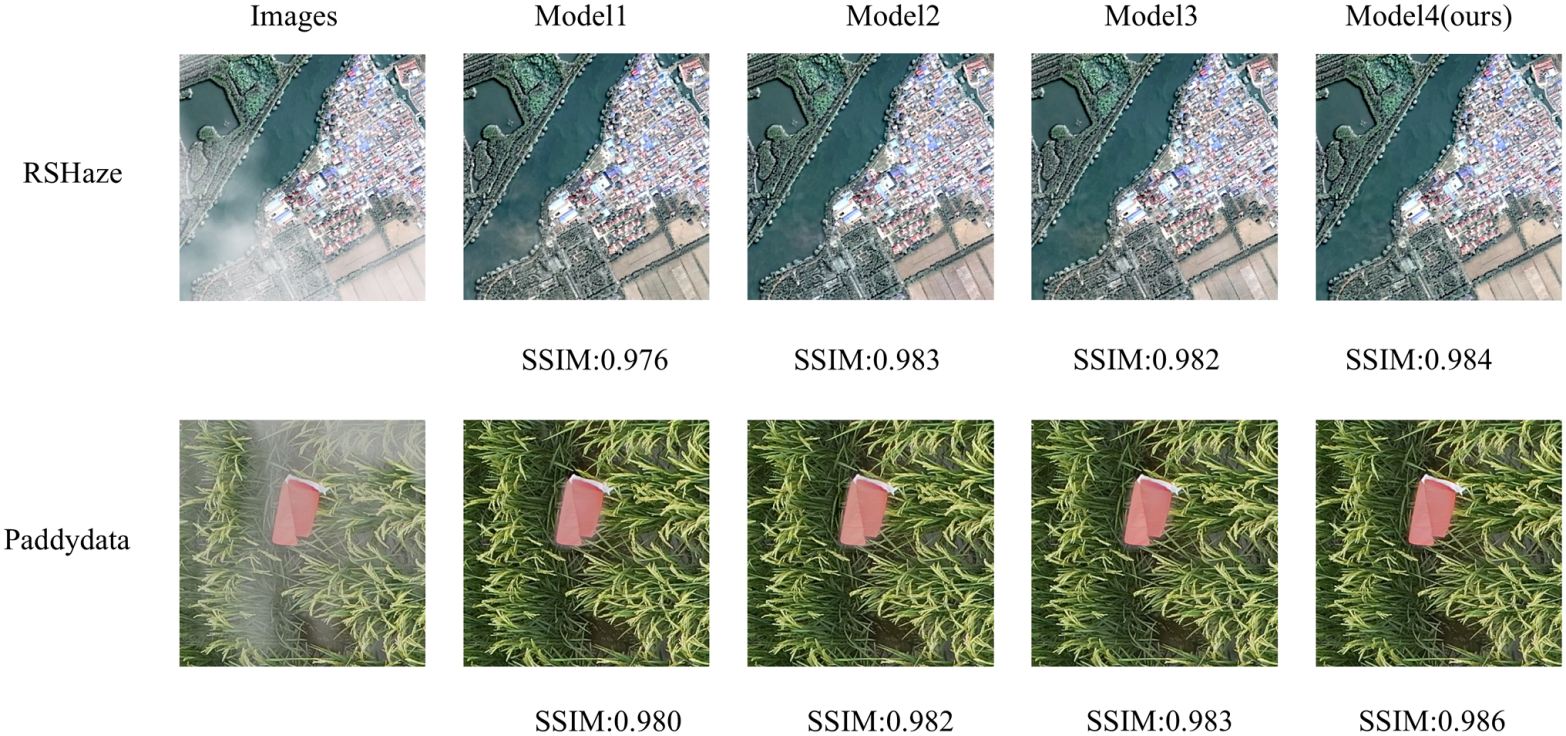
Experimental results of ablation in different modules.

This paper selected one representative image sample from each of the RSHaze and Paddydata datasets for ablation experiments, with results presented in **Figure 6**. Model2 incorporates MixConv Blocks, demonstrating superior performance to Model1 in distant scenes and hazy regions by preserving greater structural and textural detail. However, residual blurring persists in denser fog areas. Model3, featuring DFEU, exhibits enhanced stability when processing unevenly distributed haze, with more natural transitions at object boundaries. Nevertheless, clarity remains slightly compromised on minute distant structures. Model 4, combining both MixConv Block and DFEU, achieves the most balanced overall performance. It not only effectively reduces extensive haze veils but also demonstrates significant improvements in detail recovery. The image’s colour fidelity and contrast are closer to the real scene, demonstrating the effective integration of multi-scale feature extraction and adaptive path selection, thereby validating the efficacy of module combination.

The experimental results demonstrate that the MixConv Block increases the receptive field by combining depthwise separable convolution with dilated convolution. Without a significant increase in computational costs, it captures subtle differences between distant haze features and clear images. Depthwise separable convolution reduces the parameter count, ensuring computational efficiency and meeting the requirements for large-scale image processing. Meanwhile, dilated convolution, by expanding the receptive field, is better suited to handle deeper or more extensive haze layers, thus improving the precision of information in the dehazing process. The incorporation of the gating mechanism further enhances the model’s adaptability by dynamically selecting the output from different convolution paths, thereby effectively adjusting the fusion of multi-scale information. For images with varying haze intensities and distributions, the model can adaptively select the optimal feature path, thereby improving the accuracy of image detail recovery affected by haze. The application of this module in image dehazing not only enhances the ability to extract multi-scale features but also optimizes the restoration effect through an efficient weighting mechanism. Particularly in complex haze environments, it more effectively restores the true color and structure of the image. This design enhances the quality of dehazing while avoiding excessive computation and parameter redundancy introduced by traditional convolution layers. It ensures the efficiency and robustness of the entire dehazing process. DFEU predicts atmospheric light and transmission maps using dual pathways, where one path processes image information in different directions via horizontal and vertical convolutions, thereby capturing spatial feature dependencies. It adaptively learns the importance of each channel in the dehazing task, enhancing features from channels that carry critical information while suppressing irrelevant or redundant channels, thereby improving dehazing performance. The second path extracts spatial information at different scales through multi-scale adaptive pooling, incorporating a weighting mechanism to dynamically adjust feature importance at different spatial locations. Through the fusion and learning of multi-scale information, it ensures the precise restoration of haze regions of various sizes during the dehazing process, thereby improving the model’s ability to recover image details. MixConv Block extracts multi-scale haze features while retaining structural details. These enhanced features are then utilised by DFEU, which estimates transmitted light and atmospheric light through dual-branch estimation, forming a natural transition from multi-scale feature extraction to haze component estimation. The incorporation of the MixConv Block and DFEU allows for a better capture of multi-scale information in images, further enhancing the model’s ability to detect dehazing features, in the current environment of abundant computational resources, ensuring model efficiency while significantly enhancing its dehazing performance.

#### 3.3.2 Ablation experiment of DFEU

Based on the atmospheric scattering model, we proposed a Dehazing Feature Extraction Unit (DFEU) that predicts atmospheric light and transmission maps through dual pathways, establishing the relationship between hazy and clear images in feature space, and synthesizing the potential clear image features more accurately according to the physical model. To evaluate the effectiveness of DFEU, we conducted experiments comparing the FDU and PDU. Starting with gUNet+MixConv, the FDU was introduced and named Model1, the PDU was added and named Model2, and finally, the DFEU was introduced and named Model3. The experimental results are presented in **Table 2**. The “√” in the table indicates the addition of the module. From **Table 2**, it is evident that Model3, our proposed DUNet, achieved a PSNR of 37.2887 and an SSIM of 0.9933 on the RSHaze dataset. Compared to Model1 and Model2, the PSNR improved by 0.5021 and 0.2183, respectively, while the SSIM increased by 0.0005 and 0.0002, respectively. On the Paddydata foggy image dataset, the PSNR was 36.0206, and the SSIM was 0.9946. Compared to Model1 and Model2, the PSNR increased by 0.7896 and 0.2176, respectively, while the SSIM increased by 0.0005 and 0.0002, respectively.

**Table 2:** Ablation experiment results for DFEU.

Model	FDU	PDU	DFEU	RSHaze		Paddydata		Parameters/M	FLOPs/G
				PSNR(dB)	SSIM	PSNR(dB)	SSIM		
Model1	✓			36.7866	0.9928	35.2310	0.9941	3.5389	10.2435
Model2		✓		37.0704	0.9931	35.8030	0.9944	4.6154	10.6710
Model3(ours)			✓	**37.2887**	**0.9933**	**36.0206**	**0.9946**	4.1925	11.2745

The best results are marked bold.

**Figure 7:**
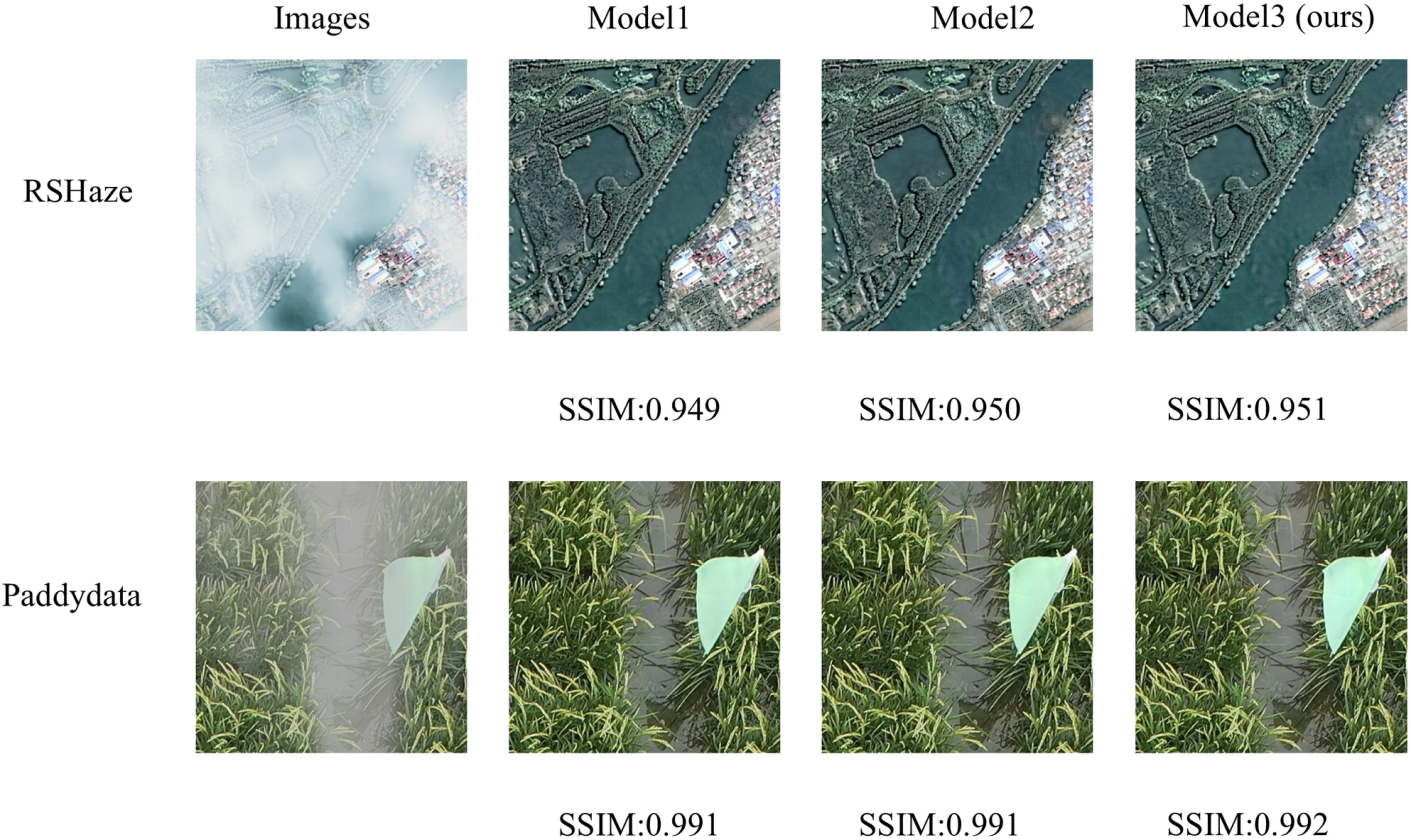
DFEU module ablation experiment results.

This paper selected one representative image sample each from the RSHaze and Paddydata datasets for ablation experiments, with results presented in **Figure 7**. Model1, failing to adequately account for the spatial non-uniformity of the transmission map, often exhibits residual haze veils in distant regions and blurred boundaries within the test images. Model2 employs dual-branch paths to model atmospheric light and transmission maps separately, yielding more natural overall colouration and improved edge clarity for foreground objects compared to FDU. However, it still exhibits insufficient detail in localised areas and extensive dense fog zones. Model 3, the proposed model in this paper, further incorporates an adaptive mechanism. In the test images, it not only restores colours more accurately in complex regions such as terrain boundaries but also maintains good clarity in fine textures and distant structures. Simultaneously, it avoids over-restoration in clear areas, achieving the optimal overall image depth and naturalness.

The experimental results show that FDU overlooks the fact that transmission maps are not uniform like atmospheric light, and using the same method to extract features for both atmospheric light and transmission maps does not lead to accurate feature representations. PDU employs dual pathways to separately extract features corresponding to atmospheric light and transmission maps, more accurately synthesizing potential clear image features and promoting information transfer and feature extraction in feature space. DFEU further enhances feature detail extraction in the dual pathways, not only adaptively adjusting the importance of feature maps but also dynamically adjusting haze intensity in various regions of the image. This results in more accurate extraction of features corresponding to atmospheric light and transmission maps, thereby improving the dehazing effect. DFEU enables the model to restore details in hazy areas more effectively, while preventing over-restoration of clear regions, thereby preserving the naturalness of the image. Compared to FDU and PDU, DFEU exhibited excellent dehazing performance on both datasets, demonstrating the success of the proposed module.

### 3.4 Comparison of experimental results of various dehazing models

To validate the performance and effectiveness of the model, we conducted a comprehensive comparison experiment using several representative models, including AODNet, DehazeNet, gUNet, AECRNet (Wu et al., 2021), GridDehazeNet (Liu et al., 2019), GCANet, PFDN, MSBDN (Dong et al., 2020), and Dehazeformer.

#### 3.4.1 Quantitative analysis

To comprehensively assess the dehazing performance of the DUNet model developed in this study, we conducted comparative experiments using existing popular models in the same experimental environment. The results of the comparative experiments are presented in **Table 3**. As presented in **Table 3**, the PSNR of DUNet on the RSHaze remote sensing dataset is 37.2887, and the SSIM is 0.9933. On the Paddydata foggy image dataset, the PSNR is 36.0206, and the SSIM is 0.9946. DUNet achieved the highest PSNR and SSIM on both datasets. Compared to Dehazeformer, the model with the best dehazing performance among the other models, the number of parameters in the model increased by 3.5051M, and FLOPs increased by 4.8337G. DUNet improved the PSNR and SSIM by 1.3505 and 0.003, respectively, on the RSHaze dataset. On the Paddydata dataset, DUNet improved the PSNR and SSIM by 0.8459 and 0.0009, respectively. Compared with other models, DUNet has obvious advantages in terms of PSNR and SSIM, proving that our model has good application potential and scalability without significantly increasing computational overhead while improving dehazing performance. The comparison and analysis of the above results clearly demonstrate that, in the image dehazing task, DUNet achieves better evaluation metrics compared to other popular models, indicating superior dehazing performance.

**Table 3:** Comparative experimental results of different models.

Model	RSHaze		Paddydata		Parameters/M	FLOPs/G
	PSNR(dB)	SSIM	PSNR(dB)	SSIM		
gUNet	33.2464	0.9894	34.7626	0.9935	0.8432	2.8304
MSBDN	35.4667	0.9908	34.1449	0.9903	28.7117	24.6672
DehazeNet	22.6331	0.9262	20.9062	0.7736	0.0092	0.5915
AOD-Net	22.4631	0.9291	19.1063	0.7531	0.0023	0.1164
GCANet	33.8754	0.9885	31.8586	0.9878	0.7021	18.5027
GridDehazeNet	33.0615	0.9895	34.3725	0.9929	0.9588	21.5598
AECRNet	31.8469	0.9847	32.1510	0.9831	2.5906	42.9314
PFDN	34.9240	0.9900	33.5383	0.9869	11.2742	50.5294
Dehazeformer	35.9382	0.9903	35.1747	0.9937	0.6874	6.4408
DUNet(ours)	**37.2887**	**0.9933**	**36.0206**	**0.9946**	4.1925	11.2745

The best results are marked bold.

#### 3.4.2 Qualitative analysis

**Figure 8:**
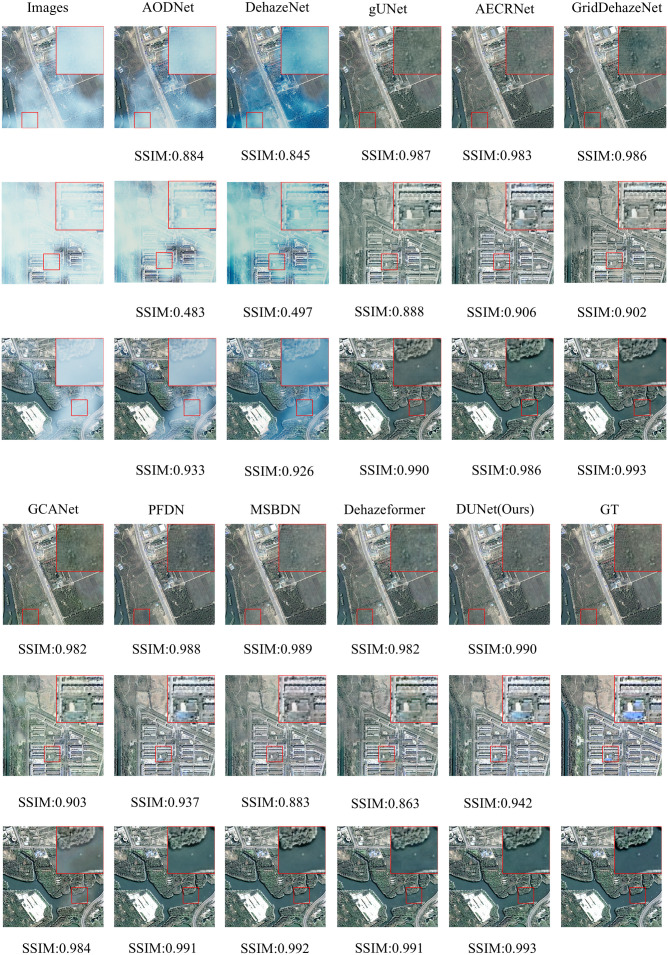
Performance evaluation of different dehazing models on the RSHaze dataset.

**Figure 9:**
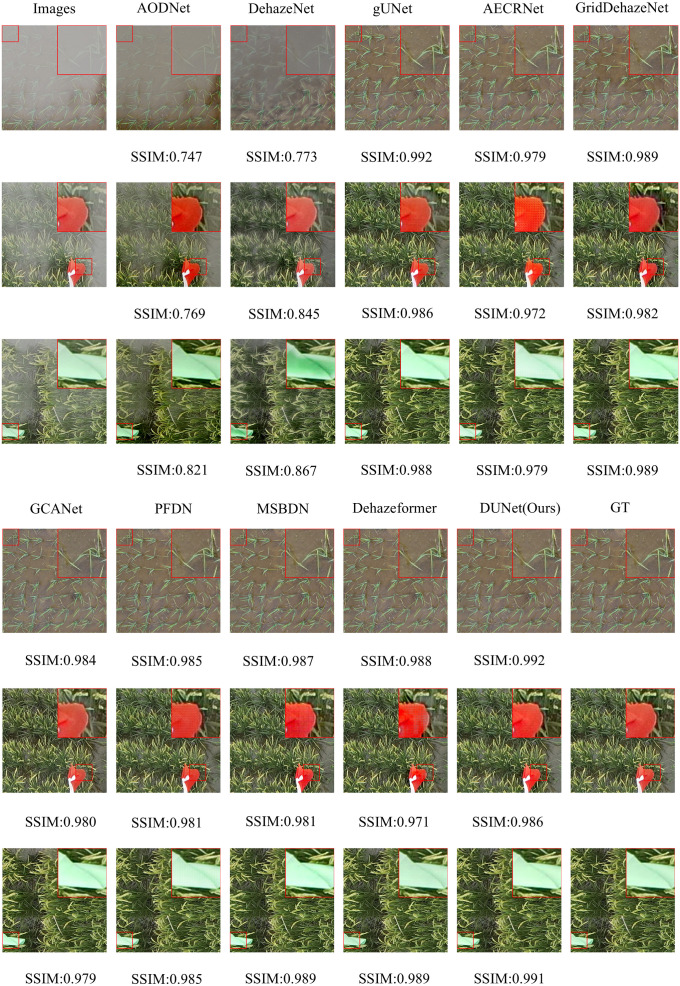
Performance evaluation of different dehazing models on the Paddydata dataset.

We selected three representative samples from the RSHaze dataset, which cover different haze concentrations in remote sensing images. Additionally, we chose three representative samples from the Paddydata dataset, containing images with varying haze concentrations collected from rice fields. These samples were qualitatively analyzed to assess the performance differences of various methods in handling segmentation accuracy, robustness, and adaptability to complex scenarios. In the selected test images, we focused on representative areas, such as color-rich regions and heavily hazy zones, and observed the dehazing performance of different models. **Figure 8** and **Figure 9** provide a qualitative comparison between DUNet and other dehazing models.

For the RSHaze dataset, early end-to-end image dehazing models, such as AODNet and DehazeNet, exhibit poor performance on remote sensing datasets. These models tend to distort when processing detailed and complex scenes, leaving significant haze in the resulting images and failing to achieve effective haze removal. Models that neglect physical feature spaces, such as gUNet, AECRNet, GridDehazeNet, and GCANet, display similar dehazing performance but still struggle with haze detail processing. As shown in **Figure 8**, these models leave residual haze in the dehazed regions, resulting in slightly hazy images with color discrepancies compared to haze-free images. This indicates that neglecting physical features impacts both haze removal and color restoration in blurred areas. PFDN and MSBDN demonstrate improved dehazing performance, however, they still suffer from artifacts at object edges and slight visual blur in the restored images. Due to its feature extraction unit, PFDN stands out in color restoration and is one of the few models that emphasize color details in the tests. The introduction of an improved Transformer Block in Dehazeformer significantly advances feature extraction, particularly at object edges, making it the second-best model after our DUNet. However, it struggles to accurately restore the original colors in bright regions obscured by haze, leading to noticeable color errors. As shown in **Figure 8**, our DUNet model outperforms all other models in dehazing, including color restoration and feature detail recovery. It not only recovers the bright regions obscured by haze but also excels in processing object details.

For the Paddydata dataset, AODNet and DehazeNet encounter the same issue. The processed paddy field images still retain some haze, which is easily noticeable, and fail to achieve effective haze removal. gUNet, AECRNet, and GCANet exhibit similar dehazing performance, however, they still show significant shortcomings in handling the edges of object details, such as the colored flag markers in the paddy fields. As shown in **Figure 9**, after processing, the edges of the flag markers still display haze features, resulting in blurry details in the output image. Furthermore, due to the residual haze, color recovery is also insufficient. GridDehazeNet, PFDN, and MSBDN further improve dehazing performance. However, they still exhibit artifacts at object edges, and the bright regions of the restored images remain slightly obscured by haze. The introduction of an improved Transformer Block in Dehazeformer leads to significant advancements in feature handling, making it second only to DUNet in terms of dehazing performance. Nevertheless, color compensation remains slightly skewed, showing deviations from the original clear images, and slight haze still lingers in certain details, such as the paddy stalks. Finally, as shown in **Figure 9**, DUNet outperforms all other models in haze removal, excelling in both object edge details and vibrant color regions. It restores sharper edges and handles detailed elements like paddy stalks and colored flags with the best performance.

### 3.5 Failure analysis and discussion

In **Figure 8** and **Figure 9**, it can be observed that the model still exhibits minor residual haze in certain areas with colour tones similar to those of haze, such as road surfaces and reflective areas of paddy fields. In such areas, where the colours of the objects are highly similar to those of the haze itself, the model may struggle to distinguish between the actual scene content and the haze components, leading to incomplete haze removal. The fundamental reason lies in the fact that, in these low-contrast areas, the model finds it difficult to accurately differentiate between the foreground and the background information obscured by the haze, thereby reducing the effectiveness of the haze removal process. This phenomenon also reflects that the current model still has room for improvement in its representation capabilities when dealing with areas with blurred edges and weakened details, particularly in terms of modelling accuracy for colour separation and structural preservation. In future research, we will enhance the model’s perception capabilities in low-contrast regions and improve its ability to distinguish between haze and background details. For example, more refined feature enhancement mechanisms or region-adaptive dehazing methods based on visual attention can be introduced to improve the model’s dehazing accuracy in regions with similar colours.

## 4 Discussion

Deep learning has demonstrated significant advantages in image restoration tasks, offering an effective approach to the problem of fog removal. However, existing methods remain prone to feature loss and edge blurring under extreme weather conditions and complex scenes. Consequently, developing efficient fog removal techniques holds considerable importance for enhancing the accuracy and stability of drone-based agricultural field monitoring.

This paper proposed a dehazing method and develops a new dehazing network to remove blurriness from remote sensing datasets and foggy paddy field image datasets. DUNet aims to fully extract clear features from the images and effectively recover visual information affected by blurriness. Specifically, the backbone network extracts multi-scale feature information from blurry images, while the MixConv convolution module captures useful information more comprehensively, improving the model’s feature representation ability when handling complex blurry images. The DFEU based on the atmospheric scattering model, establishes a mapping between the blurry and clear images in feature space through dual-path predictions, providing more precise information for the dehazing process and yielding finer dehazing features. Finally, the dynamic characteristics of the SK module enable it to flexibly adjust the feature fusion strategy under different input conditions, enhancing the model’s adaptability and robustness.

The research on image dehazing based on remote sensing and foggy paddy field image datasets demonstrates that DUNet holds significant potential in addressing challenges such as haze and blurring. The PSNR on the RSHaze remote sensing dataset is 37.2887, and the SSIM is 0.9933. On the foggy paddy field image dataset, Paddydata, the PSNR is 36.0206 and the SSIM is 0.9946. Experimental results demonstrate that, compared to other popular image dehazing models, DUNet directly establishes relationships between hazy and clear images within the feature space. This enables the model to fully leverage image physical information to extract dehazing features and effectively restore visual information impaired by factors such as haze. DUNet offers superior performance, confirming its potential and feasibility for outdoor smart agriculture dehazing tasks.

However, similar to most deep convolutional network models, DUNet relies on paired images for training. While it demonstrates strong dehazing performance on the two synthetic blurry datasets used in this study, experiments with unpaired foggy images have not yet been explored. Moreover, DUNet does not adequately balance the number of parameters with computational efficiency. Although it performs well, the inclusion of complex units increases both the number of parameters and computational demands. Furthermore, the lack of real-world foggy image datasets has long been a challenge in the image dehazing field. Most existing open-source datasets are based on clear images with synthetic haze, which undermines the authenticity of foggy datasets and negatively impacts the model’s performance.

In future research, we plan to address these limitations from three aspects. First, regarding datasets, we will collaborate with professional organizations or large laboratories to collect real foggy data, capturing paired images from the same area under both clear and foggy conditions. This will also include unpaired real foggy and clear images to ensure the authenticity and effectiveness of the dataset, which is foundational in deep learning. Second, in terms of models, developing a lightweight, high-performance image dehazing model will be a key direction, as image dehazing is primarily used as preprocessing for subsequent visual tasks. Thus, further research on model deployment and computational efficiency is necessary, focusing on lightweight yet high-performance model architectures. Additionally, incorporating better attention mechanisms and innovative feature fusion strategies will enhance the model’s adaptability to complex environmental conditions. Finally, we aim to explore unpaired image dehazing, enabling experiments to be conducted entirely based on real-world foggy images, independent of datasets. This will involve not only convolutional neural networks but also the integration of generative adversarial networks and diffusion models for future development. Through these efforts, we seek to advance image dehazing technology to meet real-world needs for high-quality image restoration, providing more accurate technical support for research and contributing to the progress of fields such as intelligent monitoring, remote sensing, and smart agriculture.

## Data Availability

The datasets presented in this study can be found in online repositories. The names of the repository/repositories and accession number(s) can be found below: https://github.com/MaiheZHao/data.
